# De novo lupus-like glomerulonephritis after pediatric non-kidney organ transplantation

**DOI:** 10.1007/s00467-021-05194-6

**Published:** 2021-07-22

**Authors:** Cristina M. Farkas-Skiles, Robert B. Ettenger, Jonathan E. Zuckerman, Meghan Pearl, Robert S. Venick, Patricia L. Weng

**Affiliations:** 1 Department of Pediatrics, Division of Nephrology, University of California Los Angeles Medical Center, Los Angeles, CA USA; 2 Department of Pathology, University of California Los Angeles Medical Center, Los Angeles, CA USA; 3 Department of Pediatrics, Division of Gastroenterology, University of California Los Angeles Medical Center, Los Angeles, CA USA

**Keywords:** Liver transplant, Intestinal transplant, Glomerulonephritis, Pediatric

## Abstract

**Background:**

We propose a novel clinically significant finding, de novo lupus-like glomerulonephritis (DNLLGN), in patients with autoantibodies and kidney abnormalities in pediatric liver transplant (LT) and intestinal inclusive transplants (ITx).

**Methods:**

We describe the clinical, serologic, and histopathologic presentation and kidney outcomes in eight patients from our center found to have DNLLGN on kidney biopsy.

**Results:**

Pediatric recipients of non-kidney solid organ transplants developed an unusual de novo immune complex glomerulonephritis with morphologic similarity to lupus nephritis. Six had isolated LT (0.9% of all pediatric LT at our center) and two had ITx (2.1% of all ITx). Five (63%) presented with nephrotic syndrome. Five patients had autoantibodies. Patients underwent kidney biopsy at a mean of 11.5 years in LT and 2.8 years in ITx after the index transplant. Biopsies demonstrated changes similar to focal or diffuse active lupus. Follow-up eGFR at a mean of 6 years after biopsy showed a mean decrease of 30 ml/min/1.73 m^2^ in all patients (p = 0.11).

**Conclusions:**

DNLLGN has not been previously recognized in this clinical setting, yet 8 kidney biopsies from pediatric recipients of LT and ITx at our center in 25 years demonstrated this finding. DNLLGN appears to be an under-reported phenomenon of clinical significance.

**Graphical abstract:**

**Figure:**
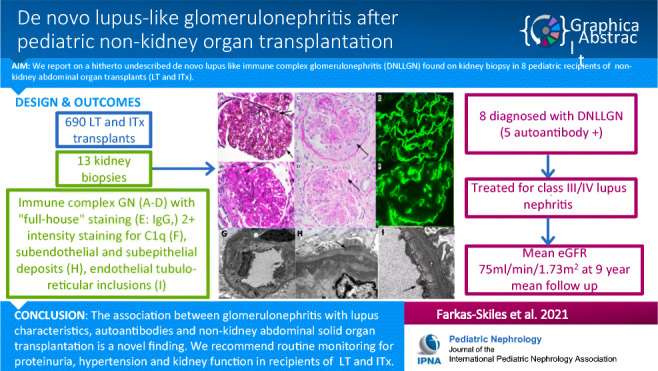
A higher resolution version of the Graphical abstract is available as Supplementary information.

## Introduction

Kidney disease is a common morbidity in patients with non-kidney abdominal organ transplants. Up to 21.3% of intestinal transplant patients develop stage 5 chronic kidney disease (CKD 5) at 60 months post-transplant [1] and up to 30% of pediatric liver recipients develop chronic kidney disease (CKD) [2]. The most frequent causes of CKD and CKD 5 in liver and multivisceral solid organ transplants are attributed to calcineurin inhibitor toxicity, thrombotic microangiopathy, nephrocalcinosis, iron overload, acute tubular injury with infections, hypertension, glomerulopathy, or polyomavirus nephropathy [1–4]. De novo glomerular disease has also been associated with progressive CKD and CKD 5 in patients with liver transplant (LT), particularly IgA nephropathy and hepatitis B- and C-associated glomerulonephritis [1, 5, 6].

De novo immune complex glomerulonephritis in adults and children has been described in a limited number of post-transplant clinical situations: with hepatitis C infection [7], after kidney transplantation [8] and idiopathic without signs of systemic lupus erythematosus [9–11]. The pattern on biopsy has been classified in two ways when full house immunofluorescent staining is seen: as (1) lupus-like glomerulonephritis, in the case where systemic signs of lupus and lupus autoantibodies develop in the future [12], or (2) idiopathic full house nephropathy (positivity in IgG, IgM, IgA, C1q, and C3) in the case where patients never develop extra-renal signs of lupus [10]. Importantly, idiopathic full house nephropathy has correlated with poorer kidney outcomes compared to patients with similar histopathology in systemic lupus [9], but this conclusion may have been confounded by differences in immunosuppressive treatments.

Isolated autoantibody elevations after LT have been thought to be of little clinical significance, particularly in the adult liver transplant population [13]. Although some prospective studies have shown that the development of autoantibodies, particularly anti-nuclear antibody (ANA), anti-mitochondrial DNA antibody (AMA), and anti-smooth muscle antibody (ASMA) are associated with de novo autoimmune hepatitis and rejection after pediatric liver transplantation [14], this conclusion remains controversial [15], and no kidney disease was studied in these populations.

Here we report on a hitherto undescribed de novo lupus-like immune complex glomerulonephritis (DNLLGN) found on kidney biopsy in eight pediatric recipients of non-kidney abdominal organ transplants. We propose that DNLLGN may be an autoimmune disease occurring in a pediatric post-transplant setting in pediatric LT and ITx patients with signs and symptoms of kidney disease such as hematuria, proteinuria, nephrotic syndrome, malignant hypertension, or acute kidney injury (AKI).

## Methods

Patients were evaluated using deidentified designations, and Institutional Review Board exemption was obtained before initiation of this study. We performed a chart review for kidney biopsies in pediatric recipients of non-kidney abdominal transplants. We included patients whose kidney biopsies demonstrated immune complex glomerulonephritis with lupus-like features. Features that qualified as “lupus like,” distinguished from other types of glomerulitis [16], included at least two of the following five pathologic features: (1) “full-house” staining, (2) ≥2+ intensity staining for C1q, (3) extra-glomerular deposits, (4) subendothelial and subepithelial deposits, and (5) endothelial tubuloreticular inclusions. Biopsies with other immune complex-mediated glomerulonephritis (e.g., IgA nephropathy) or non-glomerular pathology were excluded. A kidney pathologist (JZ) blinded to the patients’ clinical course reexamined the available light microscopy, immunofluorescence, and electron microscopy studies for the purpose of this study and scored available slides by the 2017 ISN/RPS classification of lupus nephritis. If any component of the biopsy was not available for review, the relevant data was gleaned from the review of the microscopic description in the original pathology report. Glass slides from patients 3 and 8 were not available for re-review. A single investigator (CFS) reviewed the patients’ electronic medical records for past medical history, medications, laboratory values, comorbidities, final diagnosis by their nephrologist (ongoing SLE, remission, recovery, or deceased), and kidney measures. Data were stored using REDCap software and outputs were deidentified [17]. Comorbidities including thrombosis, surgical complications, infections, episodes of rejection, and malignancy history were collected. Estimated GFRs (eGFR) were used to assess kidney function, as measured GFRs were not universally available. eGFRs were calculated based on age at time of incident biopsy. The bedside Schwartz equation was used for patients <18 years old [18] and the CKD-EPI equation [19] was used for patients ≥ 18 years old. Study end was defined as last available follow-up to May 1, 2018. The maximum eGFR for any patient was set at 89 ml/min/1.73 m^2^, so as not to overestimate GFR for low muscle bulk or malnutrition. All patients’ medications were standard of care by the institutional protocol for their era; e.g., prednisone was discontinued in a majority of patients 6 months after transplant.

## Results

### Pre-biopsy clinical characteristics

From January 1993–May 2018, 690 pediatric LT and 96 ITx were performed at UCLA Mattel Children’s Hospital. Thirteen of these patients (9 LT and 4 ITx) manifested clinical changes in urine studies and underwent kidney biopsy. Of this group, eight patients had kidney biopsies showing lupus-like features, including six with LT (66% of biopsied patients; 0.9% of all LT) and two with ITx (50% of biopsied patients; 2.1% of all ITX). The remaining five patients who had biopsies were diagnosed with drug-induced lupus, C1q nephropathy, and low grade immune complex mesangial proliferative glomerulonephritis.

The patient demographics are summarized in Table 1. All patients were less than 4 years of age at time of transplantation (mean 1.5 ± 1.3 years). The majority (63%) were Hispanic. The most common primary disease was primary biliary cirrhosis (63%). All six LT cases were complicated by portal vein or hepatic artery thrombosis in the immediate postoperative period; three required re-transplantation (patients 2, 4, 5). Four patients had a history of autoimmune disease including psoriasis (patient 7), arthritis (patient 3), and de novo autoimmune hepatitis (patients 3, 4, 6). One patient (patient 6) had prior history of post-transplant lymphoproliferative disorder (PTLD) and was in remission at time of kidney biopsy.

**Table 1:** Patient characteristics

Pt	Gender	Ethnicity/race	Organ Txp (number of Txp)	Age at Txp (yr)	Age at Bx (yr)	Follow up time from Bx to study end (yr)	Primary diagnosis	Previous auto-immune and thrombotic disease
1	F	Hispanic/Asian	Liver, SB pancreas (1)	3.2	4.6	3.5	Short gut syndrome, prematurity, IFLD	IVC thrombus
2	F	Hispanic	Liver (2)	0.6	14.0	6	Biliary atresia	Portal vein thrombosis
3	M	Hispanic	Liver (1)	0.6	11.3	5	Biliary atresia	Arthritis dAIH, portal vein thrombosis
4	M	Hispanic	Liver (3)	3.6	17.3	9	Biliary atresia	dAIH, hepatic artery thrombosis
5	F	Not Hispanic	Liver (2)	0.7	9.6	15	Biliary atresia	Portal vein thrombosis
6	F	Hispanic	Liver (1)	0.6	11.9	5.5	Biliary atresia	dAIH, acquired protein C deficiency, portal vein thrombosis, hepatic artery thrombosis
7	M	Not Hispanic/White	Liver, SB(1)	1.8	6.0	8	Gastroschisis, short gut syndrome, prematurity, IFLD	Psoriasis
8	M	Not Hispanic/Black	Liver (1)	0.6	14.4	0.5	Cryptogenic cirrhosis, sickle cell SC	Portal vein thrombosis

Abbreviations: *Bx* biopsy, *dAIH* de novo autoimmune hepatitis, *IFLD* intestinal failure asocial liver disease, *IVC* inferior vena cava, *SB* small bowel, *Txp* transplant, *Yr* year

Patient clinical presentation and outcome after the diagnosis of clinical kidney disease can be found in Table 2. Kidney biopsies occurred at a mean time of 11.5 ± 2.5 years after LT and 2.8 years after ITx. The mean patient age at biopsy was 11.1 ± 4.3 years. Indications for kidney biopsy included nephrotic syndrome (n = 6) and persistent proteinuria/hematuria (n = 2). Two patients with nephrotic syndrome also had severe extra-renal autoimmune manifestations, including polymyositis, pericardial effusion, pleural effusion, hemolytic anemia, and leukopenia. Less severe clinical symptoms in the other patients included mild peripheral edema and hypertension. Four patients had a documented infection for which they were treated when they presented with urine abnormalities and/or changes in eGFR, which led to biopsies. The infections were bacteremia (2), urinary tract infection (1), and hemophagocytic lymphohistiocytosis (HLH), for which the triggering organism was not identified. Seven patients were on immunosuppression at the time of clinical presentation and kidney biopsy including tacrolimus (75%), cyclosporine, mycophenolate mofetil (38%), and prednisone (25%). One patient was off all immunosuppression.

**Table 2:** Clinical history and outcome

Pt	Circulating serologies and complement	Clinical presentation	Kidney biopsy time post Txp (years)	Immuno-suppressive medications at time of Bx	Infection at presentation	Outcome
1	*Positive: ANA, CL IgG & IgA* Negative: B2G IgG, B2G IgM, B2G IgA, Sm, RNP, SSA/SSB Ab, ANCA	H,P	1.4	MMF, Tac, Pd	Bacteremia (coagulase neg *Staph aureus*)	Remission
2	Negative C3, C4	H,P;	13.3	Tac	No	Active SLE
3	*Positive dsDNA, low C3, low C4* Negative ANA, ANCA, CL IgG & IgM	NS, polymyositis Pericardial effusion Pleural effusions hemolytic anemia, leukopenia	10.7	Tac	Bacteremia (methicillin-sensitive *Staph aureus*)	Active SLE
4	Negative: ANA, anti-LKM, dsDNA, ANCA, ASMA	P	13.7	MMF, Tac, Pd, Muromonab-CD3	No	Remission
5	n/a	NS	9.0	Cyc, Pd	No	Remission
6	*Positive: ANA, CL IgG & IgA, dsDNA, B2G IgA, low C3, low C4* Negative Thyroglobulin Antibody, ANCA, Sm, RNP, SSA/SSB	NS	11.3	None	UTI (*Enterococcus*)	Deceased
7	*Positive: ANA, CL IgG, low C3, low C4* Negative: CL IgM, CL IgA, ANCA, Sm, B2G IgG, B2G IgM, B2G IGA, RNP, SSA/SSB	NS, fevers, pericardial effusion, pleural effusions, neutropenia, PRES/seizures	4.2	MMF, Tac	HLH (inciting infection unknown)	Active SLE
8	*Positive: dsDNA* Negative: ANA B2G IgG, B2G IgM, B2g IgA, CL IgG, CL IgA, CL IgM, DNAseB, SSA/SSB, C3, C4	NS	13.8	Tac	No	Remission

Abbreviations: *ANA* antinuclear antibody, *ANCA* antineutrophil cytoplasmic antibodies, *DNAseB* antideoxyribonuclease-B antibody, *anti-LKM* anti-liver-kidney microsome type 1 antibody, *ASMA* antismooth muscle antibody, *B2G* beta-2-glycoprotein antibody, *Bx* biopsy, *CL* Cardiolipin, *Cyc* cyclosporine, *dsDNA* anti-double-stranded DNA antibody, *H* hematuria, *HLH* hemophagocytic lymphohistiocytosis, *MMF* mycophenolic acid, *NS* nephrotic syndrome, *P* proteinuria, *Pd* prednisone, *Pos* positive, *Pt* patient, *Neg* negative, anti ribonucleoprotein antibody SLE systemic lupus erythematosus, *anti-Smith* antibody Sm, *SSA/SSB* antiSSA/antiSSB antibodies, *Tac* tacrolimus, *Txp* transplant, *UTI* urinary tract infection

### Kidney biopsy findings

**Figure 1:**
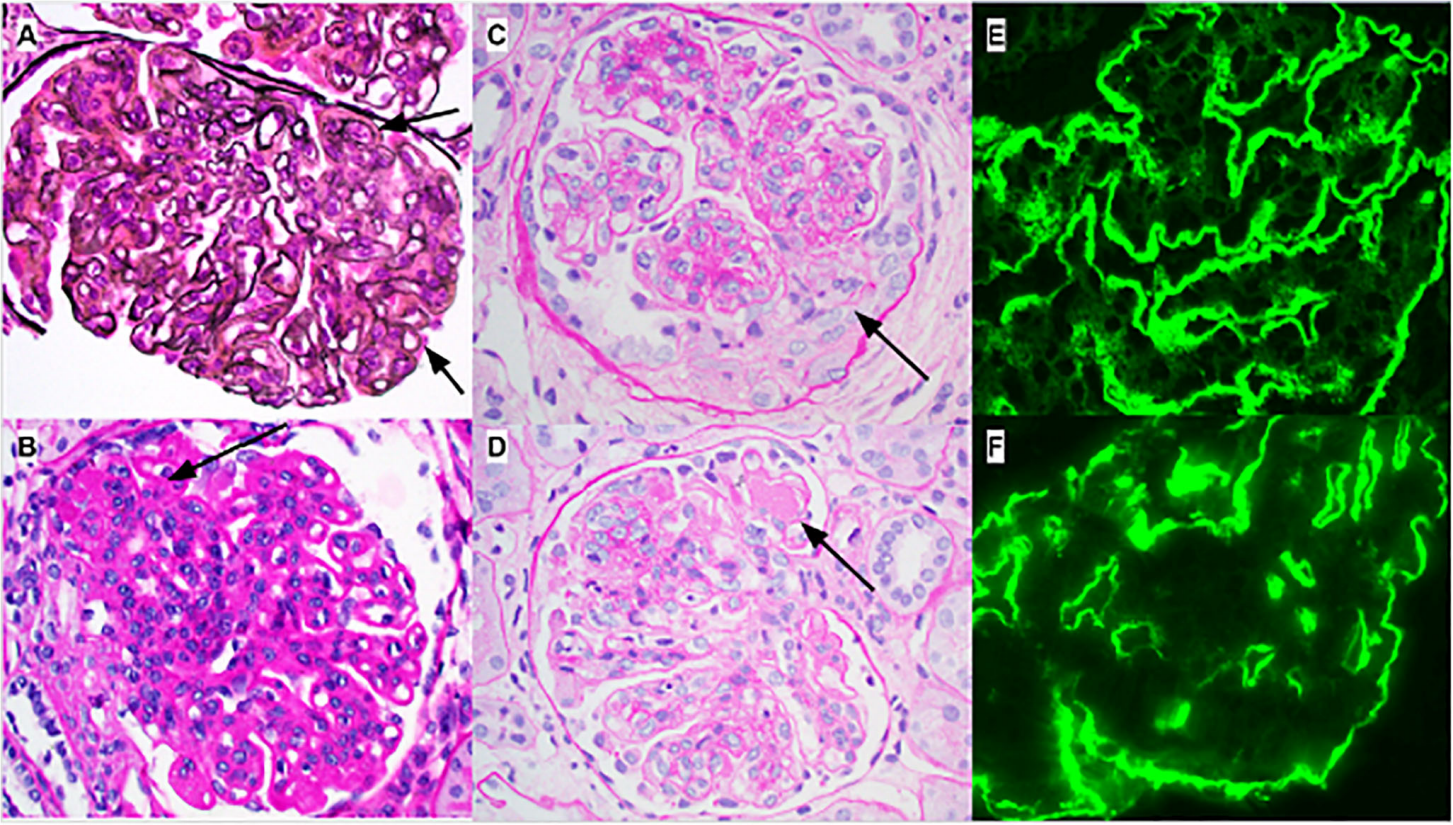
Representative light and immunofluorescence microscopy findings on biopsy. Glomeruli from patients 6 (**A**–**B**) and 7 (**C**–**D**) demonstrating a membranoproliferative glomerulonephritis (MPGN) pattern of injury represented by mesangial and endocapillary hypercellularity and prominent capillary loop double contours (arrows panel **A**). Rare segmental sclerosis was present (arrow panel **B**) in some cases. Patient 7 showed segmental cellular crescents (arrow panel **C**) and large endocapillary deposits (arrow panel **D**). Immunofluorescence staining for IgG (**E**) and C1q (**F**) from patient 6 are shown and demonstrate bright interrupted linear glomerular capillary loop and granular mesangial staining (**A**: Jones silver stain; **B**–**D**: periodic acid Schiff stain). All images are 400×

Seven of eight patients demonstrated an active histological lupus-like glomerulonephritis (Table 3); the eighth patient (patient 4) had significant chronicity. The majority demonstrated diffuse active features (n = 5) and the remaining had focal active features (n = 3) corresponding to 2017 ISN/RPS lupus nephritis class IV and class III, respectively. The features of activity include endocapillary hypercellularity (n = 7) with focal endocapillary neutrophils (n = 4), focal large fuchsinophilic deposits (wire loops/hyaline pseudothrombi) (n = 5), and mild interstitial inflammation (n = 6) (Figure 1A–D). Patient 7 showed severe activity with crescents and fibrinoid necrosis (Figure 1C); patient 7 had the most severe pathology at presentation. The average activity index was 4.9 ± 3.5. Mesangial hypercellularity was present in all biopsies. Some biopsies also showed mesangial sclerosis (Figure 1B).

**Table 3:** Native kidney biopsy results

Patient	1	2	3^a^	4	5	6		7		8^a^
SLE class	III	IV	IV	III	III	IV		IV		IV
Biopsy #						1	2	1	2	
Total glomeruli (# global sclerotic)	24 (0)	23 (1)	17(1)	21 (8)	33 (8)	24 (3)	62 (2)	41 (2)	30 (3)	54(2)
Activity index	4	5	4	0	2	8	4	4	12	5
Chronicity index	1	3	4	5	4	1	1	1	1	1
Light microscopy										
Glomerular involvement	Focal	Diffuse	Diffuse	Focal	Focal	Diffuse	Diffuse	Diffuse	Diffuse	Diffuse
Cellular crescents	-	-	-	-	-	-	-	-	Focal (20%)	-
Fibrinoid necrosis	-	-	-	-	-	-	-	-	Focal (23%)	-
Endocapillary hypercellularity	Focal	Diffuse	Segmental	-	Focal	Diffuse	Diffuse	Diffuse	Diffuse	Diffuse
Neutrophils/karryoexic	Focal	-	-	-	-	Focal	Focal	-	Focal	-
Wire loops/hyaline hyper-thrombi	-	Focal	Focal, rare	-	-	Diffuse	-	-	Focal	-
Segmental sclerosis/fibrous crescents	Focal	Focal	Focal	Focal	Focal	Focal	Focal	-	-	Focal
Capillary loop double contours	Focal	Diffuse	Focal, rare	-	-	Diffuse	Diffuse	-	+	-
Membranous features	-	-	-	-	-	-	+	-		+, rare
Mesangial hypercellularity	+	+	-	+	+	+	-	+		+
Interstitial inflammation	Mild	Mild	-	Mild	Mild	Mild	-	Mild		-
Interstitial fibrosis/Tubular atrophy (%)	-	10–15%	20%	<15%	10%	-	-	-	<15%	-
Arterio/arteriosclerosis	-	Moderate	Mild	Mild	Minimal	-	-	-	-	-
Immunofluorescence										
IgG^b^	2+	2+	3+	2+	1+	3-4+	Trace-1+	1+	Trace	2+
IgA^b^	-	1+	2+	Trace	Trace	1-2+	-	2+	3+	3+
IgM^b^	3+	3+	3+	Trace	2+	3+	1–2+	Trace	-	3+
C1q^b^	1+	2+	3+	1+	Trace	3+	1+	2+	Trace	2–3+
C3^b^	-	2+	2+	Trace	Trace	2+	1-2+	1+	2+	3+
Kappa, Lambda	-, -	2+, 2+	3+, 3+	Trace, 1+	Trace, Trace	2+, 2+	1+, 1–2+		2+, 2+	3+, 3+
Electron microscopy										
Mesangial deposits	+	+	+	+	+	+	+	+	+	+
Subendothelial deposits^b^	+	+	+, large	+	+	+, numerous	+	+	+	+
Intramembranous deposits	-	-	-	-	+	+, rare	+	-	-	-
Subepithelial deposits^b^	+, segmental	+, segmental	-	+	+	+, rare	+, rare	+, rare	+, rare	+, seg-mental
Foot process effacement (mild, moderate, severe)	Minimal	Extensive	Extensive	Moderate	Moderate	Extensive	Moderate	Extensive	Moderate	Mild
Tubuloreticular inclusions^b^	+	-	-	-	-	+		+	+	-
Increased mesangial lipid deposition	+	-	+	-	+	-	-	-	-	+
Double contours	+	-	+	-	+	-	-	+	+	+

^a^Patients 3 and 8’s slides were not available for re-review

^b^Distinguishing pathologic features of lupus nephritis from nonlupus glomerulonephritis

Most biopsies demonstrated minimal to mild chronicity including focal global glomerulosclerosis and focal segmental glomerulosclerosis/fibrous crescents, but at most, only mild interstitial fibrosis/tubular atrophy. The average chronicity index was 2 ± 1.6. All cases showed capillary loop double contours either by light or electron microscopy. One case had focal and segmental membranous features by light microscopy. Mild to moderate arteriosclerosis and arteriolar sclerosis were present in three biopsies

**Figure 2:**
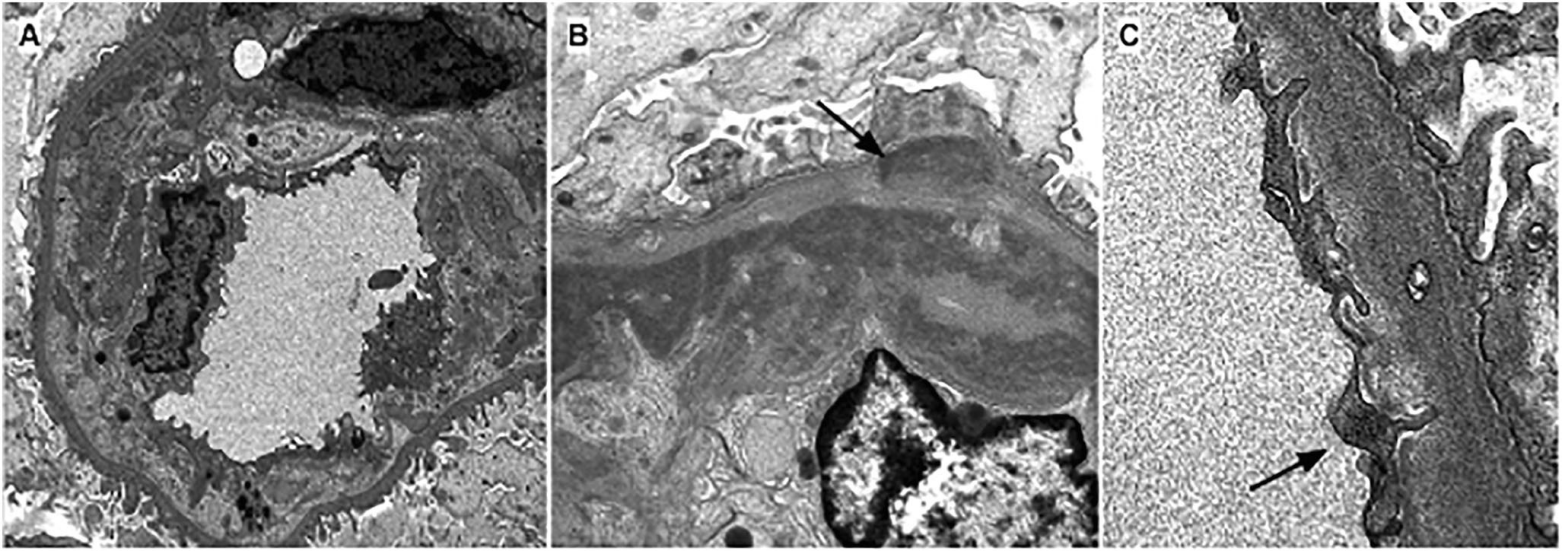
Representative electron microscopy findings on biopsy. All patients exhibited varying degrees of double contour formation and subendothelial deposits. **A** Representative images of a glomerular capillary loops with prominent double contour formation (patient 1) represented by subendothelial neomembrane and cell process interposition associated with subendothelial deposits. **B** High magnification view of a double contour (patient 6) demonstrating granular electron dense immune complex deposits in subendothelial and rarely subepithelial (arrow) locations. **C** Glomerular capillary loops showing a cytoplasmic tubuloreticular inclusion within an endothelial cell (patient 7) (arrow)

Six patients had “full-house” immunofluorescence (IF) staining in at least one biopsy. All cases had deposits in mesangial, subendothelial, and subepithelial deposits by either IF (Figure 1E–F) or EM analysis (Figure 2A–B). At least two cases also showed intramembranous deposits. Rare deposits with fibrillary substructure were present in two biopsies. The biopsies with focal activity tended to have less intense IF staining compared to those cases with diffuse glomerular involvement. Three cases had tubular reticular inclusions (TRI), illustrated in Figure 2C. There was also increased glomerular extra-cellular lipid accumulation in five biopsies, which is a finding often seen in the setting of chronic liver disease. There were no extra-glomerular deposits present in any biopsy by IF or EM. There were no instances of lupus vasculopathy, vasculitis, or thrombotic microangiopathy. No biopsies showed features of calcineurin inhibitor toxicity.

### Serologic evaluation

Serologic evaluation including C3, C4, ANA, anticardiolipin IgG and IgM, anti-beta-2-glycoprotein IgA, IgG, and IgM (B2G), anti-double-stranded DNA (dsDNA), myeloperoxidase antibody, proteinase 3-antibody, anti-liver-kidney microsome type 1 antibody (anti-LKM1), AMA, ASMA, and ribonucleoprotein antibody was available for seven patients; the missing data were collected at another institution and not available for review. Seventy-one percent of these patients had at least one positive autoantibody (Table 2) or complement marker including dsDNA (3), ANA (2), antiB2G IgA (1), anticardiolipin IgG (2), low C3 (2), and low C4 (2). Two patients had a negative serologic work up.

### Post-biopsy outcomes

**Figure 3:**
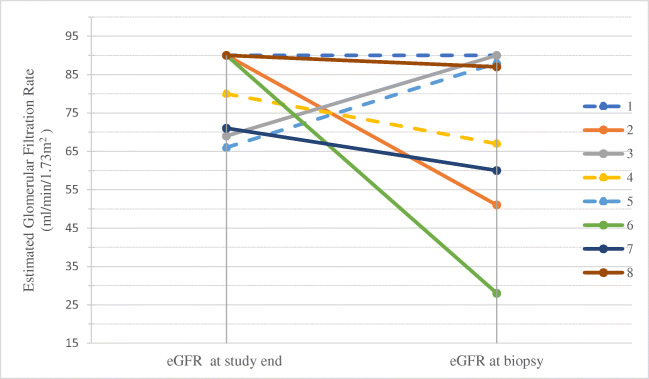
Estimated GFR decline from presentation to study end. eGFR shows a mean decrement in eGFR of 30 ml/min/1.73 m^2^ in all patients (p = 0.11). Dashed lines represent patients with focal DNLLGN and solid lines represent patients with diffuse DNLLGN

Patients were subsequently treated as a class III/IV lupus glomerulonephritis with first line of induction with steroid pulse and mycophenolate. Response to treatment was variable. The patients with focal DNLLGN all achieved clinical remission after induction without relapses, whereas the patients with diffuse glomerular disease had only partial treatment responses requiring continued therapy. Two patients had post-treatment biopsies. The morphologic features in the follow-up biopsies were similar, although the foot process effacement had improved with treatment in patient 6 but did not improve in patient 7. Four patients had ongoing CKD stage 2–4 at study end, and one expired from unexpected cardiac arrest with liver failure and CKD 5D, 68 months after kidney biopsy. Mean estimated GFR at study end was 75 mL/min/1.73 m^2^, corresponding to a mean decrease of 30 mL/min/1.73 m^2^ at the 9-year mean study end time post-biopsy; this was not a statistically significant decrease (p = 0.11) although a trend was evident as shown in Figure 3. Estimated GFR tended to be lower at follow-up in patients whose biopsies had diffuse DNLLGN, with a mean eGFR decreased from 81 to 63 mL/min/1.73 m^2^, noted with solid lines in Figure 3. Patients with focal DNLLGN showed stable eGFR 78–81 mL/min/1.73 m^2^ at study end, noted with dashed lines in Figure 3. The mean eGFR decrement was larger in ITx patients (mean decrease of 44 mL/min/1.73 m^2^) compared to LT (mean decrement of 33 ml/min/1.73 m^2^).

## Discussion

We describe 8 patients in a single pediatric transplant center who were recipients of LT or ITx and who developed de novo lupus-like immune complex glomerulonephritis (DNLLGN.) This is a novel finding, and the association between glomerulonephritis with lupus characteristics, autoantibodies, and non-kidney abdominal solid organ transplantation has not been previously reported.

To our knowledge, no other studies evaluating kidney biopsies after non-kidney organ transplantation have reported cases of lupus-like glomerulonephritis [2]. Two prior cases occurring in the setting of liver transplantation have been reported as “lupus nephritis” in a single-center case series including one pediatric LT recipient and one adult LT recipient [5], diagnosed based on mesangial proliferative changes and full house immune complex deposits on IF. However, this study did not include serum autoantibodies, extra-renal clinical symptoms, complete histopathology description, or specific declines in eGFR. As such, a robust clinical association could not be hypothesized. The diagnosis of DNLLGN in our cohort was based upon the patients’ clinical trajectories as well as rigorous histologic evidence that was a characteristic of lupus nephritis, including multi-compartment immune complex deposition (i.e., co-existence of mesangial, subendothelial, subepithelial, and intramembranous deposits), large wire loop/hyaline pseudothrombi-type deposits, full house immunofluorescence staining, and the presence of tubuloreticular inclusions. Some features characteristic for lupus nephritis, namely extra-glomerular deposits, were not present in any case. Importantly, patients did not consistently have extra-renal clinical manifestations of SLE suggesting that the observed DNLLGN is predominantly a kidney-limited lupus-like glomerulonephritis, and not necessarily a manifestation of systemic collagen vascular disease. However, one patient had ongoing extra-renal disease at study end. The severity of AKI at presentation differed among patients. Taken together, our study represents the largest and most definitive case series of these findings to date and the first to report such changes in recipients of ITx.

Prior to this study, autoantibodies which have been reported to be of importance after LT are primarily ANA, ASMA, AMA, and anti-LKM1 antibody, while others think these are not significant. A prospective study showed the clinical significance of these autoantibodies in pediatric LT without history of autoimmunity, with increasing risk of chronic rejection and de novo autoimmune hepatitis [14]. Autoantibodies to endothelial antigens have also been associated with transplant complications in pediatric LT recipients [20, 21]. It is possible that the pathogenesis of autoantibodies seen in our patients with DNLLGN is similar to these other autoimmune phenomena observed following transplantation, but more studies are necessary to establish this. Interestingly, all LT with a lupus-like presentation were complicated by thrombosis of some kind; over 30 years, the percentages of operations with these complications were 7.5% for hepatic artery thrombosis and 3.2% for portal vein thrombosis [22]. But no LT had evidence of lupus anticoagulant on serology during the laboratory work up prior to transplantation.

In the setting of organ transplantation, autoimmunity could be stimulated by medications, specifically calcineurin inhibitors, vascular injury [23], or infections [24]. Interestingly, three patients presented with abnormal urine studies in the context of bacterial infections and one with HLH, of which the triggering organism was not identified. Bacterial products interact with toll-like receptors, which can provoke the production of potentially deleterious antibodies [25]. Autoimmune manifestations have also been reported with viral infections including Epstein bar virus, cytomegalovirus, and human parvovirus B19 infections [24, 25].

We found declining eGFR over a long follow-up period, in agreement with prior literature for the ITx and LT populations. Decrements in eGFR observed in these patients were likely multifactorial, due both to DNLLGN and multiple AKI episodes and drug toxicities, all of which accompany non-kidney abdominal organ transplant. It was of interest to note that patients with diffuse proliferative DNLLGN appeared to have a more pronounced decrement in eGFR than did patients with focal proliferative changes.

Our study has some important limitations including its small size and limited data collected. We were unable to obtain serologic data from one patient and pathology slides for re-review in two patients, due to the fact that the workup was performed at another institution. This study is a retrospective chart review in a single center, which inherently cannot show correlations or true prevalence. Many patients were found to have proteinuria incidentally during a work up for infection, and a prospective cohort would be better at identifying risk factors for autoantibody production and progression of CKD or glomerular disease in pediatric LT and ITx patients. We describe a decline in eGFR over time but do not have a matched cohort group to compare the decline of eGFR so we cannot say if this is the natural history after transplantation or directly related to DNLLGN. What we describe as a lupus-like presentation could be immune-mediated glomerulonephritis of post-infectious or unclear etiology or true de novo SLE in some cases. A prospective study could better differentiate between these etiologies and the role of DNLLGN in reduced kidney function. Finally, it is not clear from our data whether pediatric LT and/or ITx patients are more likely to develop DNLLGN changes than are adult patients. Furthermore, at our center, which is a large and experienced transplant center in both children and adults, DNLLGN has not been noted in adult patients, but rather only in pediatrics.

This is the most definitive report of DNLLGN in patients with LT and ITx and the largest series of its type in patients of any age. The long follow-up time in a single center is a further strength of the study. We recommend routine monitoring for proteinuria and kidney function in recipients of ITx and LT, and a nephrology referral for co-management of hypertension and abnormalities on urinalysis. In the presence of these or other signs and symptoms of changes in kidney measures, the differential diagnosis should include DNLLGN. A kidney biopsy and a full autoimmune serological workup may be necessary [5, 26]. Additional studies including multicenter surveillance of proteinuria, CKD, and glomerular diseases in LT and ITx are needed to measure the true incidence of this interesting phenomenon.

## Abbreviations

AMA: anti-mitochondrial DNA antibody
ANA: anti-nuclear antibody
anti-LKM1: anti-liver-kidney microsome type 1 antibody
ASMA: anti-smooth muscle antibody
B2G: beta-2-glycoprotein
CKD: chronic kidney disease
CKD 5: stage 5 chronic kidney disease
dAIH: de novo autoimmune hepatitis
DNLLGN: de novo lupus-like glomerulonephritis
dsDNA: anti-double-stranded DNA
eGFR: estimated glomerular filtration rate
EM: electron microscopy
GFR: glomerular filtration rate
HLH: hemophagocytic lymphohistiocytosis
IF: immunofluorescence
ITx: intestinal inclusive transplant
LT: liver transplant
MMF: mycophenolic acid
NS: nephrotic syndrome
Pd: prednisone
SLE: systemic lupus erythematosus
TRI: tubular reticular inclusions
Txp: transplant
UTI: urinary tract infection
